# P-872. Culture Shock: Antibiotic Use in Invasive Kidney Stone Surgeries

**DOI:** 10.1093/ofid/ofaf695.1080

**Published:** 2026-01-11

**Authors:** Adam Zimilover, Sammy Cheng, Erika Orner, Mei H Chang, Inessa Gendlina

**Affiliations:** Montefiore Medical Center - Albert Einstein College of Medicine, Bronx, NY; Albert Einstein College of Medicine, Bronx, New York; Montefiore Medical Center, Bronx, New York; Montefiore Medical Center, Bronx, New York; Albert Einstein College of Medicine, Bronx, New York

## Abstract

**Background:**

Genitourinary procedures carry a risk of postoperative infections, with serious systemic infections occurring in up to 5% of patients. Guidelines from the Infectious Diseases Society of America (IDSA) and the American Urological Association (AUA) recommend preoperative urine culture screening and targeted prophylactic antibiotics, with an emphasis on single-dose perioperative prophylaxis. Longer pre- or postoperative courses are not routinely recommended as the impact on infection rates and patient outcomes remains incompletely understood. Additionally, real-world adherence to these recommendations and its impact on rates of postoperative bacteremia remain poorly characterized.

**Methods:**

Retrospective review of all ureteroscopies and percutaneous nephrolithotomies performed at our institution between January 1, 2022 and December 31, 2024 was completed to identify any postoperative bacteremia episodes within 30 days of the procedure. Prior cultures and antibiotic administration data were reviewed to assess whether pre- and peri-operative antibiotics aligned with guideline recommendations and provided appropriate coverage for known culture results.

**Results:**

Of the 2825 distinct procedures reviewed, 31 cases of confirmed bacteremia were identified within 30 days of the procedure. Twelve cases involved organisms that matched preoperative culture screens. Only 8 cases of bacteremia were identified as preventable based on mismatch between antibiotics chosen and previously known cultures. Patients with inappropriate antibiotic selection during procedure were up to three times more likely to develop postoperative infectious complications (surrogate of blood culture collection).

**Conclusion:**

Periprocedural antibiotic administration is recommended to prevent postprocedural complications including bacteremia in patients undergoing PCNL or ureteroscopy. Infections are caused by a diverse group of organisms and patient-specific microbiomes as well as the local antibiogram should be used in an individualized approach to perioperative antibiotic selection. We support a “shorter is better” paradigm in not extending antibiotic use beyond perioperative doses, while individualizing care for high-risk patients.

**Disclosures:**

All Authors: No reported disclosures

